# Which Gait Parameters and Walking Patterns Show the Significant Differences Between Parkinson’s Disease and Healthy Participants?

**DOI:** 10.3390/bios9020059

**Published:** 2019-04-25

**Authors:** Sana M Keloth, Rekha Viswanathan, Beth Jelfs, Sridhar Arjunan, Sanjay Raghav, Dinesh Kumar

**Affiliations:** 1 Biosignals Lab, School of Engineering, RMIT University, Melbourne, VIC 3000, Australia; s3614093@student.rmit.edu.au (S.M.K.); s3427376@student.rmit.edu.au (R.V.); beth.jelfs@rmit.edu.au (B.J.); sridhararjunan@gmail.com (S.A.); 2 Dandenong Neurology, Melbourne, VIC 3175, Australia; sanjay.raghav@rmit.edu.au

**Keywords:** Parkinson’s disease, gait, variance, IMU

## Abstract

This study investigated the difference in the gait of patients with Parkinson’s disease (PD), age-matched controls and young controls during three walking patterns. Experiments were conducted with 24 PD, 24 age-matched controls and 24 young controls, and four gait intervals were measured using inertial measurement units (IMU). Group differences between the mean and variance of the gait parameters (stride interval, stance interval, swing interval and double support interval) for the three groups were calculated and statistical significance was tested. The results showed that the variance in each of the four gait parameters of PD patients was significantly higher compared with the controls, irrespective of the three walking patterns. This study showed that the variance of any of the gait interval parameters obtained using IMU during any of the walking patterns could be used to differentiate between the gait of PD and control people.

## 1. Introduction

Parkinson’s disease (PD) patients suffer gait disturbances, which are a major cause of disability, falls, reduced mobility and quality of life [1,2,3]. The walking style of PD patients is characterized by short shuffling steps and slowness in movement. Gait assessment is important in the diagnosis and monitoring of the disease. Gait is one of the measures for the Unified Parkinson’s Disease Rating Scale (UPDRS) and is scored by clinical observations to determine the severity of disease and efficacy of treatment. However, this is a subjective test, and there is a need for quantifiable gait analysis to study PD patients [4]. 

To address this need, many studies have quantified the differences in the gait of PD and control participants [5,6,7]. In the past the applications of such investigations were very limited due to the high cost of gait laboratories, the growth of microelectronics and sensor technology, which has given the potential for the use of Inertial Measurement Units (IMUs) with wireless capabilities to measure gait parameters in an inexpensive and portable manner. This requires the identification of the measurable parameters that best describe the difference between PD patients and controls. 

People with normal gait, when walking on an even surface, have long-range correlation between their strides, their inter-stride variability is insignificant, and the gait is rhythmic [8]. On the other hand, PD patients have high variability [8,9] and variable fractal properties [10] of the inter-stride intervals. The study by Osamu et al. [11] observed comparable results when investigating the spectral properties of stride variability with the range of the power spectrum four times larger for PD patients than healthy subjects. Similarly, Krishnan et al. [12] performed statistical analysis of the gait parameters and found higher gait variability in PD patients. Significantly higher inter-swing variability [10] and an increase in double-support stance intervals were also reported in PD patients [13]. Thus, from literature, it is evident that there are a number of gait parameters that can be used to differentiate between PD and controls. 

Gait interval measurement has the advantage of being recorded by IMU, and these parameters have been considered for the diagnosis of PD [5,10,14,15]. However, these are influenced by a number of compounding factors, such as the height, weight and age of the person [16,17,18]. The severity and duration of the disease can also influence the walking style of PD patients. The walking conditions and pattern of the path can also influence gait parameters [19,20,21]. While earlier studies have reported differences between PD and controls, numbers of these factors such as the walking pattern [22,23] and its relation to an age-matched control [24,25] have not been considered. 

Some walking patterns such as turning have been found to be affected even in the early stages of PD, with increased turning arcs [26], time to complete the turn [27,28] and a larger number of steps taken to complete the turn [29]. The number of steps and peak speed during turning significantly differed among control, mild PD and severe PD patients [30]. It has been suggested that turning is more likely to cause functional impairment than straight walking since turning involves inter-limb coordination for the re-orientation of the body towards a new direction, balance relation between posture and gait and modification of walking patterns [31,32]. The main consequence of turns in PD is lateral falls, which can result in an eight-fold increase in hip fractures compared with falls during straight walking [33,34]. Thus, it is very important to evaluate the turning ability in PD and to investigate the effect of gait periods across different turns. During the UPDRS screening, neurologists observe their patients during the turn phase of their walks, but this is subjective and has not been quantified. 

Researchers have proposed indices to quantify the variability in gait by an index, called variability index [35]. One of the main indexes used for this purpose is the Gait Phase Quality Index (GPQI), which shows how a PD gait pattern deviates from the normal healthy subject [36]. It is the Euclidean distance, in a space of gait phase distribution, between the point determined by the gait phases percentage of the examined stride and the point determined by the average distribution of gait phases among healthy subjects. A GPQI value close to 0% represents a gait pattern very similar to the healthy groups [35]. 

This study investigated the effect of age, PD and walking patterns on gait intervals with the aim of identifying walking pattern parameters that showed large differences between PD and the control. The mean and variance of four parameters were considered: stride interval, swing interval, stance interval and double support interval. Experiments were conducted where the participants performed three walking patterns: straight line, U-turn and turning around a point during a single walking trial. The group differences of the gait parameters between PD patients, age-matched controls, and young healthy participants for the three walking patterns were obtained.

There are two novelties of the study which have the potential to make a significant difference to the clinical assessment of PD patients. The first is that this study has shown that both, straight line walking and turning are suitable for the evaluation of PD patients, and hence either could be used. Thus, the practice of making the patient perform complicated turns [34,37,38] is not required for such a study.

The second novelty is that this study has shown that by the use of IMU placed on the legs of the patients and measuring the gait period variance [39,40,41], it is possible to identify PD patients while the patient performs simple walking. This has the potential to be used for population-based screening for early diagnosis of the disease. 

Another important finding of this study is that it showed that the gait variance of these parameters only showed the difference between PD and controls, irrespective of their age. Thus, while there are significant differences in the gait parameters between young and old, the variability due to age was not significant.

## 2. Materials and Methods

The experimental protocol was approved by RMIT University Human Research Ethics Committee (BSEHAPP 22-15). The aim and experimental protocol were explained to the participants and their written informed consent was obtained before the start of the experiment. The study investigated the gait data of 72 subjects: 24 with Parkinson’s disease referred to as PD, 24 age matched controls referred to as OL and 24 young controls referred to as YL. All PD patients were recruited from the PD outpatient clinic at Dandenong Neurology, Melbourne, Australia, while the OL subjects were from multiple aged-care facilities and recreation facilities and YL were recruited from RMIT University through appropriately located posters. The PD subjects were excluded from the study if there was any clinically observed or self-reported skeletal injuries, neurological, musculoskeletal diseases other than PD and UPDRS > 50. An individual’s UPDRS score >50 indicated that the patient had severe PD symptoms which were considered high-risk and unsuitable for the experiment by the human experiments ethics committee. The OL subjects were recruited to match the age distribution and gender of the PD patients approximately. The age-matched control (OL) participants were with no reported or observable PD symptoms. To confirm the suitability of the participants as controls, they were assessed according to the guidelines of the motor examination section of the Unified Parkinson’s Disease Rating Scale (UPDRS), Hoehn and Yahr (H & Y) scale and their self-assessment. They were excluded if there was any sign of PD, clinically observed or self-reported skeletal injuries, neurological, musculoskeletal diseases. 

All PD patients were in their ON phase of the medication cycle. The number of participants in the experiment was based on the power calculation in order to achieve a statistical power of 80% [42]. The age group of participants considered for the study was 20–80 years. There was no gender bias in this study. 

Participant’s demographic data, medical history, psychiatric history, current medication and PD history (duration, symptoms, previous medication time, progression) was collected and de-identified for their privacy. They were assessed according to the guidelines of the motor examination section of the Unified Parkinson’s Disease Rating Scale (UPDRS), the intensity and disability scales from the Unified Dyskinesia Rating Scale (UDysRS), Hoehn and Yahr (H & Y) scale and the cognitive test from the Montreal Cognitive Assessment (MOCA). Table 1 shows the clinical characteristics of the three groups.

### 2.1. Data Recording

A wireless Trigno IMU (Delsys, Boston, USA) system was used for the data recording. The system had three channels each for acceleration, rotation and magnetic field and one for surface electromyogram (EMG). The EMG electrodes were active electrodes with an inter-electrode distance of 20 mm and bandwidth of 20–450 Hz. The maximum wireless operating range of the sensor was 20 m. The sampling rate of the EMG signals was 1111.11 samples/second, of the accelerometer and gyroscope signals 148.14 samples/s and of the magnetometer signals was 74.07 samples/s. The axis of the IMU was aligned with the anatomical axis of the leg, with the vertical axis of the sensor mounted parallel to the tibia bone [43,44].

**Figure 1:**
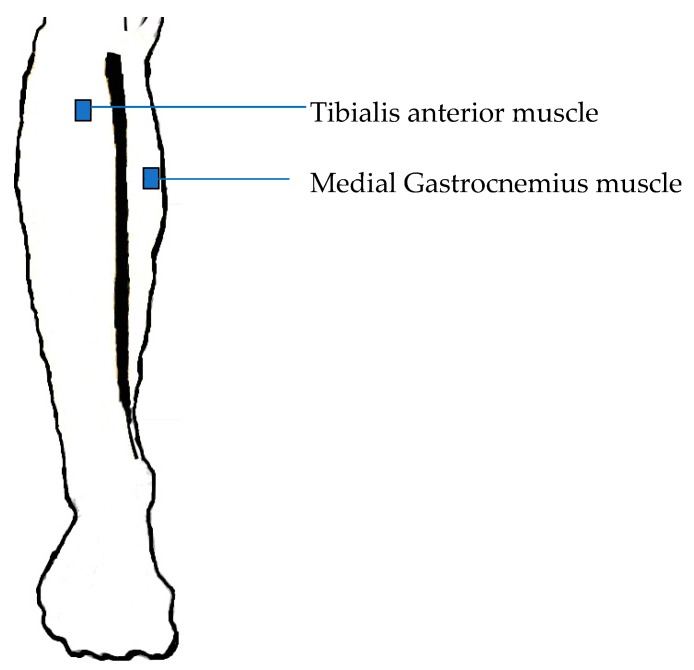
Location of the sensor on the right leg [48].

The IMUs were placed on the Medial Gastrocnemius muscle (MG) and Tibialis Anterior (TA) muscles of the left and right legs as shown in Figure 1 and the positioning was based on the SENIAM recommendation [45]. The sensor placed in the TA muscle was used to compute the gait intervals, which was considered the best location to study gait events [46]. The acceleration and angular velocity curves in the Medio-Lateral (ML) axis of the IMU sensor placed in the TA muscle was further used for the calculation [47].

### 2.2. Experimental Protocol

**Figure 2:**
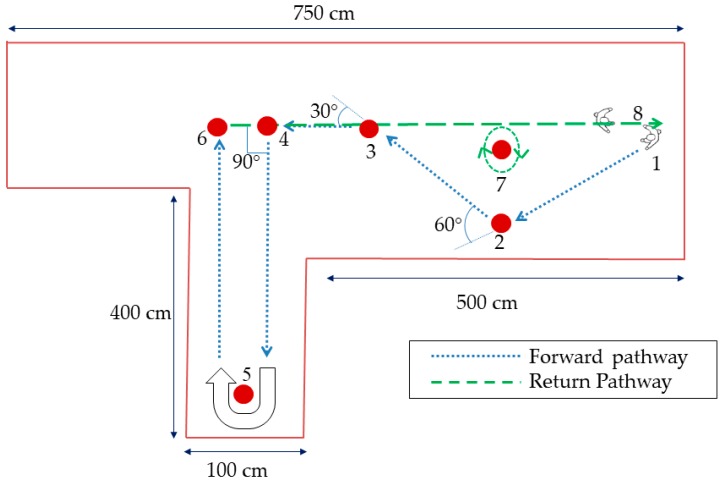
Walkway. **1.** Start position, **2.** 60° turn, **3.** 30° turn, **4.** 90° turn, **5.** U-turn, **6.** 90° turn, **7.** Turn around a point, **8.** Turn from spot, **1.** Start position.

The protocol required the participants to walk along a path marked on the floor with white markers and as shown in Figure 2. A 600 mm diameter obstacle was placed at points 5 and 7 to guide the participants to perform a U-turn and turn around a point respectively. All participants were encouraged to familiarise themselves with the path and equipment before starting the recording. Assessments were video-recorded and taken for reference. 

### 2.3. Pre-processing of the Signal

The IMU recordings were pre-processed to remove noise and offset. The offset in the recordings was removed using MATLAB. Secondly, the noise in the accelerometer and gyrometer was corrected using a second order bandpass Butterworth filter with a cut-off frequency 0.01 Hz–20 Hz. 

### 2.4. Turn Identification

**Figure 3:**
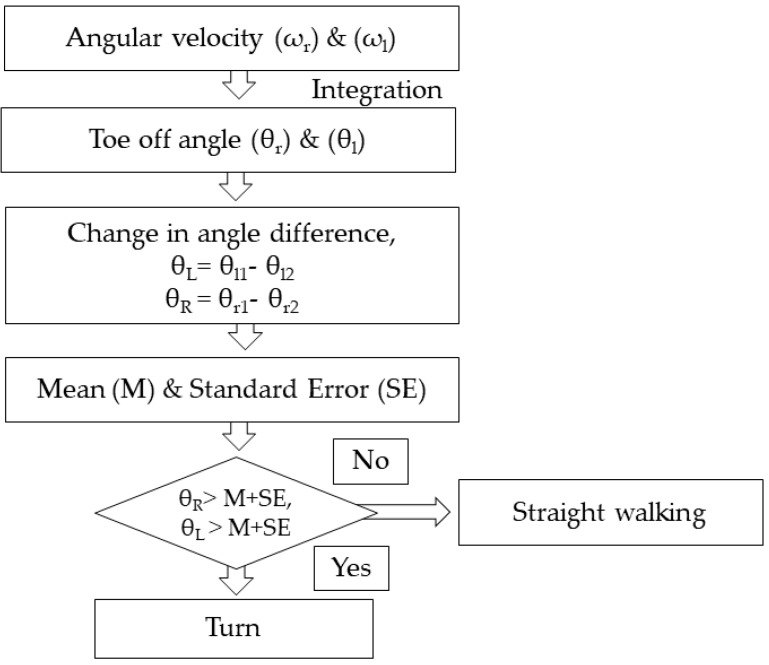
Flowchart to distinguish the turns from straight walking.

A change in the direction of walking is defined as a turn. To identify turns, the heel strike angular velocity in the Medio-Lateral (ML) axis of the IMU sensor placed in the TA muscle was considered for the study. The heel strike angle was calculated by the trapezoidal integration of the angular velocity curve of both right and left limb. Finally the change in difference of the heel strike angle of the same limb was used to categorize straight walking and turns [49]. There was small but statistically insignificant difference between the right and left side (*p* < 0.05) for all subject groups and for further analysis only the dominant right leg was considered. The selection of the dominant side was based on the questionnaire. Out of 24 PD and 24 YL, all were right dominant and for OL excluding one, all were right dominant. The flow chart describing the procedure to distinguish turns from straight walking is given in Figure 3.

**Figure 4:**
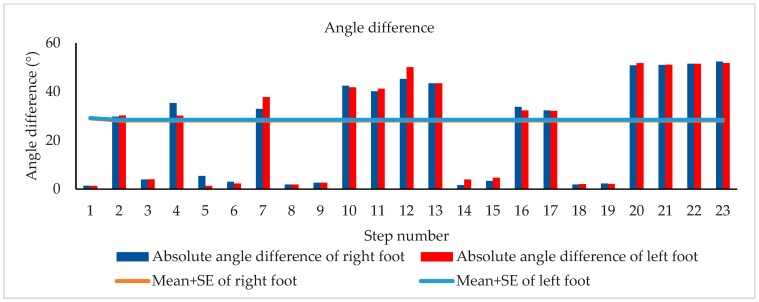
Angle difference of one subject during walking.

Figure 4 shows the absolute angle difference of one subject during walking. The turn was identified when the difference between the absolute angles of either right or left foot was greater than M + SE of the respective foot [49].

### 2.5. Gait Phase Identification

**Figure 5:**
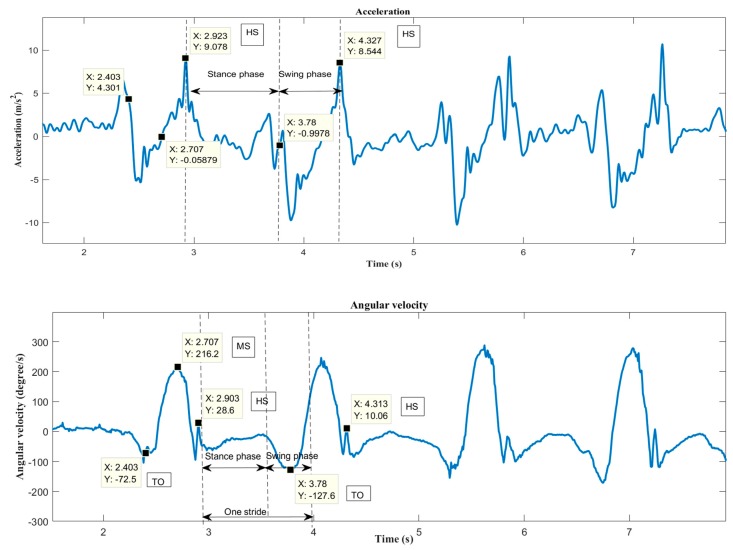
Acceleration and angular velocity curves showing HS, TO and MS of one stride.

A gait cycle is defined as the difference between the times of two consecutive heel strikes of the same leg. The heel strike is the moment when the heel touches the ground and is identified by the highest peak in the acceleration curve [50]. PD patients have smaller heel strike angles when compared to the healthy cohort [51], and the heel strike can be confused with the start of the swing phase. To avoid false heel strike detection, the gyroscope signal was also used to detect the end of the swing phase (mid-swing) represented as a peak in the gyroscope signal. The corresponding maximum peak in the accelerometer signal then represents the heel strike [52]. Figure 5 shows the pre-processed acceleration and angular velocity curves in the medio-lateral (ML) axis of the IMU sensor placed in the TA muscle, depicting the HS (heel strike), TO (toe-off) and MS (mid swing) phases of gait. 

### 2.6. Gait Feature Extraction

The following gait parameters were calculated from the right leg:

- Number of steps during turn (steps).
- Total turn duration (s).
- Cadence = total number of steps/total turn duration (steps/min) for turns and for straight walking the total turn duration was the total duration of straight walking.
- Stride duration– Time from HS to HS of same foot (s).
- Stance duration–Time from HS to TO of same foot (s).
- Swing duration–Time from TO to HS of same foot (s).
- Double support duration–Time from right HS to left TO + Time from left HS to right TO (s)
- The variance of gait intervals was computed using the coefficient of variance (*σ*), as it was found to be the most common method in analyzing the gait fluctuation [53]. The *σ* for each gait interval was calculated as the ratio of standard deviation of the gait parameter to the mean of the gait parameter. The variance of the stride interval, swing interval, stance interval and double support interval were represented as σ_st_, σ_sw_, σ_sta_ and σ_d__s_ respectively. Similarly, the mean of the stride interval, swing interval, stance interval and double support interval were represented as µ_st_, µ_sw_, µ_sta_ and µ_d__s_, respectively.
- Gait Phase Quality Index (GPQI) was calculated using the following formula [35]:
  (1) $$\mathrm{GPQI}\;=\;\sqrt{\sum_{i\;=\;1}^{2}(FDS_{PD}-mFDS_{OL})+(SS_{PD}-mSS_{OL})+(SDS_{PD}-mSDS_{OL})+(SW_{PD}-mSW_{OL})}$$
  where *FDS_PD_, SS_PD_, SDS_PD_, SW_PD_* represented the percentage gait phase of PD, *mFDS_PD_, mSS_PD_, mSDS_PD_, mSW_PD_* represented the average value of percentage gait phase of OL. The GPQI calculation was computed for PD, to access the effect of gait phase distribution and represented by GPQIPDO and for OL computed as the gait phase distribution of OL, which differed from the OL average and represented by GPQIOL. Similarly, a calculation was performed with respect to the average value of the percentage gait phase of YL. The corresponding GPQI value for PD was represented by GPQIPDY and compared with GPQIYL. The GPQI was calculated for each subject and the average values were plotted.

### 2.7. Statistical Analysis

The Shapiro–Wilk Test was performed to check for normal distribution of the data, as it gave the highest power of distribution when compared to other similar tests [54]. The data was not normally distributed and all the statistical significances of the group-based difference was obtained using the Kruskal–Wallis (KW) test which is a non-parametric test, recommended for comparing between multiple independent groups which have no normal distribution of data [55]. When significant differences were found, a Bonferroni’s test was performed [35]. Cadence, total turn duration, number of steps, σ_st_, σ_sw_, σ_sta_, σ_d__s_, µ_st_, µ_sw_, µ_sta_ and µ_d__s_ were analyzed using KW test when checking for group difference. The Wilcoxon test was performed to assess the difference within a group. The significance level was set as 0.05 for all the statistical tests performed.

## 3. Results

**Figure 6:**
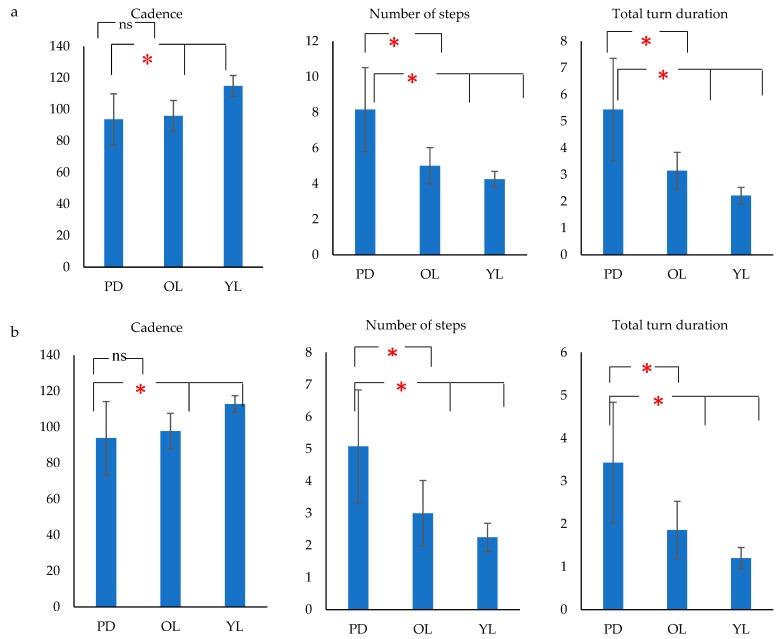
Bar charts showing mean cadence, number of steps and total turn duration for PD, OL and YL subjects for (**a**) U- turn and (**b**) Turn around a point (*p (Significance) < 0.05, ns (non-significant)).

Figure 6 shows that there was an age associated trend of reduced cadence, increased number of steps and total duration for the turning task. It was also seen that there was a significant difference between these parameters among PD patients and age-matched controls. The cadence was statistically insignificant between the groups for straight walking and hence not reported. 

**Figure 7:**
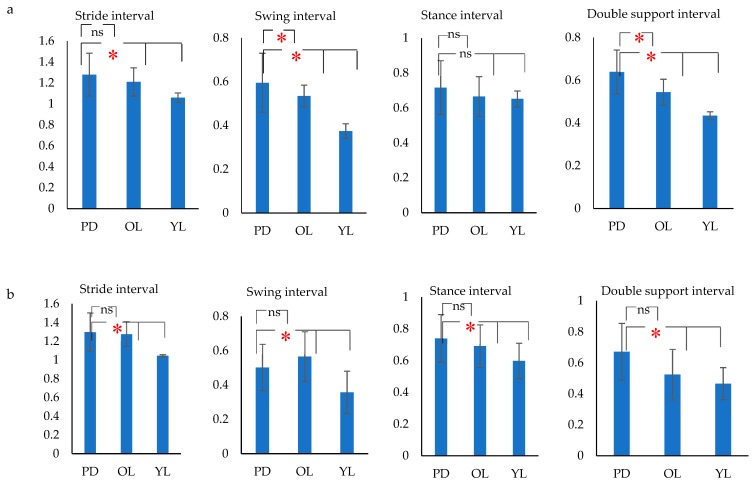

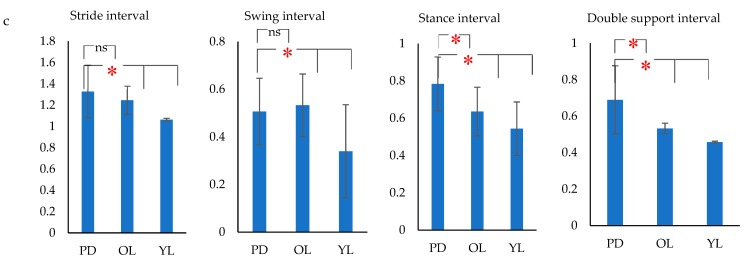
Bar charts showing mean (SD) stride interval, swing interval, stance interval and double support interval for PD, OL and YL subjects for (**a**) Straight walking (**b**) Turn around a point (**c**) U- turn (*p (Significance) < 0.05, ns (non-significant)).

From Figure 7, it can be seen that while some of the parameters did not show a significant difference between the groups, there was a significant difference for some of the gait intervals between PD, aged matched control and young participants for the three patterns of walking– straight, turn around a point and U-turn. It was also observed that even when the group difference between the mean values was significant, this was of the order of 10 to 15%. 

**Figure 8:**
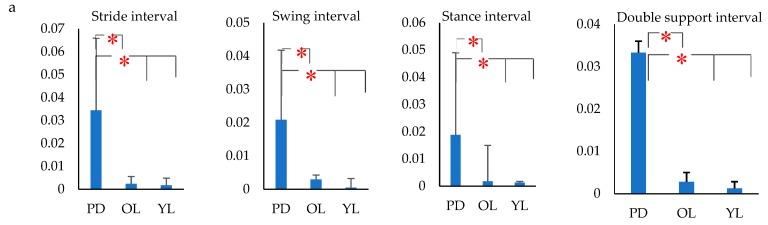

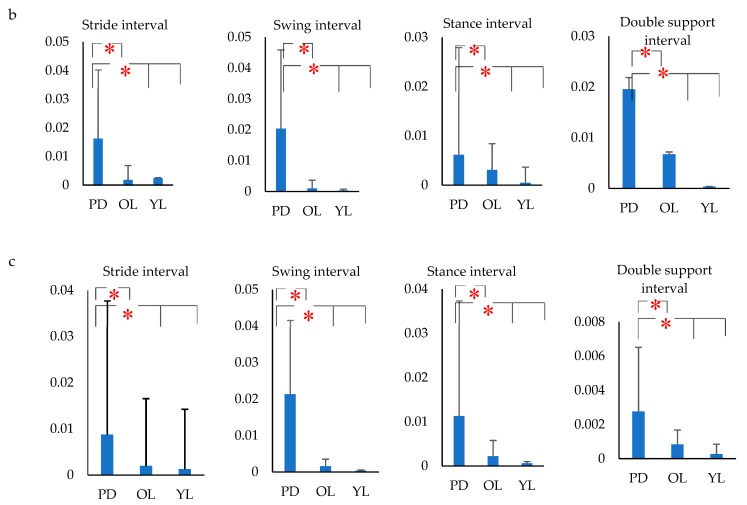
Bar charts showing variance (SD) of stride interval, swing interval, stance interval and double support interval for PD, OL and YL subjects for (**a**) Straight walking (**b**) Turn around a point (**c**) U- turn (*p (Significance) < 0.05).

From Figure 8 it is observed that there was a significant increase in the variability of all the gait interval parameters for all the three activities, and this was more pronounced when compared with the mean values (Figure 7). From Figure 6, Figure 7 and Figure 8, it is seen that the variance in all the four gait intervals, i.e., stride, swing, stance and double support, showed the highest difference between PD and control, irrespective of the control being age-matched or young, and this was the case for all the three gait tasks. This indicated that variance rather than the values of the gait interval parameters were suitable for differentiating between PD and control subjects, and may be suitable for the diagnosis of PD. It was also seen that the age-associated change in the variance was small when compared with the increase due to disease. 

Table 2 shows the *p* value obtained by comparing the mean and coefficient of variance of gait intervals for straight walking with U-turn and turn around a point separately. It was seen that the variance of gait intervals significantly differed for all the gait intervals for straight walking when compared to turns, except for the swing interval variance. While, age-matched control (OL) subjects didn’t show any significant difference in gait interval variance based on the walking pattern, except for double support interval variance. It was also seen that young control (YL), showed insignificant difference in gait interval variance based on the walking pattern. Furthermore, the mean of the gait intervals was statistically insignificant based on the walking pattern, except for a few intervals as tabulated in Table 2. 

**Figure 9:**
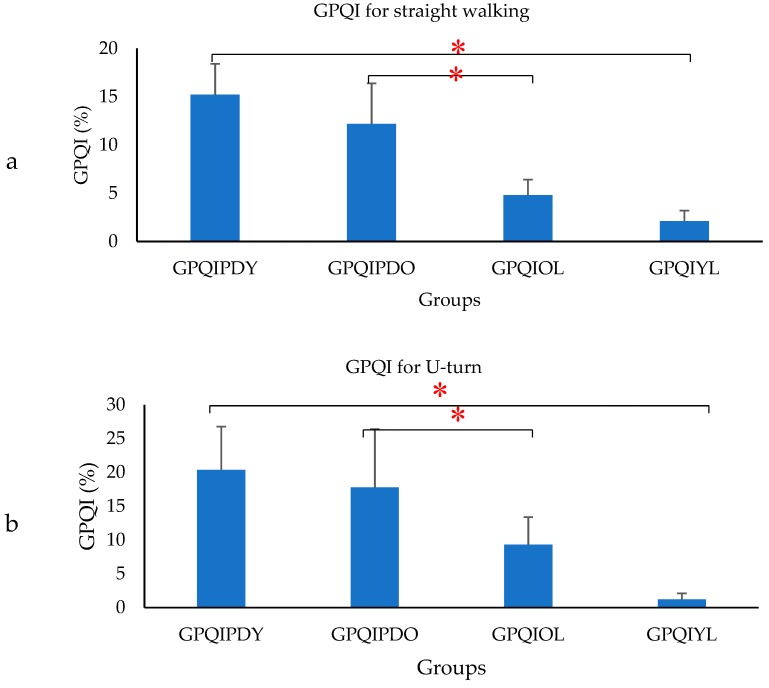

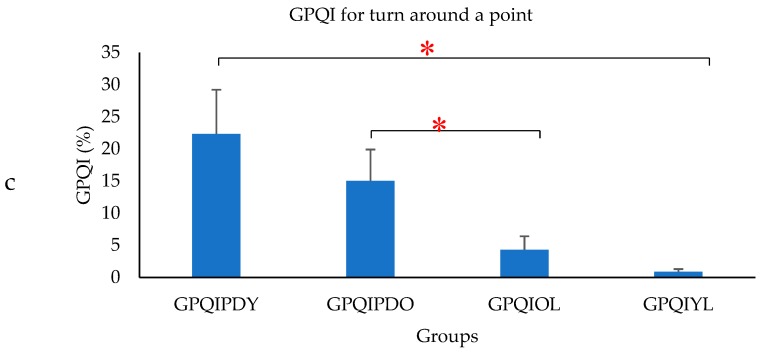
Bar plot showing the mean (SD) GPQI for (**a**) straight walking, (**b**) U-turn and (**c**) Turn around a point (*p (Significance) < 0.05).

Figure 9 shows that there was a significant difference between GPQIPDO and GPQIOL and similarly between GPQIPDY and GPQIYL for each of the walking patterns. The GPQI value was statistically insignificant based on the walking pattern and hence not reported.

## 4. Discussion

Gait variability can arise due to intrinsic and extrinsic causes [56]. Variability in gait recordings may arise due to the type of walking surface, level of ambient light or even due to instrumentation error [57,58,59]. Other causes are inherent to the person such as neurological, metabolic and musculoskeletal health and injury. 

This study investigated the group difference of gait parameters– cadence, number of steps and total turn duration during U-turn and turn around a point. The results showed that for the turning task, the cadence decreased and the number of steps and total turn duration increased with aging. The results showed that the gait of healthy young people was rhythmic and variation in their gait parameters was small when compared with the older cohort, or PD patients. The decrease in cadence, increase in number of steps and total turn duration in the age matched control were in line with the literature [60,61], where it has been reported that the variations in gait may be due to supraspinal and central pattern generators [62] or the difference in time scale inputs arriving at the brain from visual, vestibular and mechanoreceptors in the feet [63]. Aging is an internal variation that can cause changes in the natural bipedal locomotion [64,65]. Our results showed that cadence did not show significant differences between PD (non-freezers) and OL, while the number of steps and total turn duration had a significant difference between the groups. While [28] has shown that there was no significant difference between cadence of PD freezers, non-freezers and controls during a 180 degree turn but, [66,67] found that cadence increased significantly in freezers when compared to non-freezers and control during 180- and 360-degree turns. Both the studies above were performed in an OFF-period of medication while our study was with patients in their ON-state of medication. Literature [68,69] has shown the independence of cadence in PD ON and OFF period of medication when compared to the control. This study has confirmed that, cadence did not significantly differ between PD and OL, while the number of steps and total turn duration was statistically different between the PD and OL while performing a turn. Cadence also didn’t have a statistically significant difference between the groups for straight walking and hence was not reported. 

This study also investigated the group differences of gait intervals between PD, aged matched control and young control during straight walking, taking a U-turn and turn around a point. The results showed that there were significant differences between PD and age matched control, and between the young and older cohort for most of the parameters. However, the largest difference between PD and control, irrespective of age, was seen in the variance measured as the coefficient of variance of the gait interval rather than the mean values of the parameters and was observed for all the three walking tasks that were investigated. This shows that while there are age-associated changes to the gait parameters the difference in variance between PD and control is significant, even without considering the age, and the difference was much greater than all other parameters. In the case of PD patients, irrespective of any of the three aforementioned walking tasks, there is a significant decrease in the ability to generate gait rhythm. This supports the works of Redgrave et al. [70] who found that PD patients lose their habit control systems in the basal ganglia which leads to a greater dependence on voluntary control of ‘habitual’ activities such as walking due to which there is greater variability. Literature [23,71,72] also show that the presence of neurological disorders such as in PD have major effects in increasing gait variability. The increase in stride variability in gait was a unique indicator of the inability to produce gait rhythm [73,74,75] and risk of falls [7,76]. Loss of dopamine in the substantia nigra leading to the excessive inhibition of the basal ganglia loop leads to the loss of habitual patterns [70] associated with walking and also causes rigid movement and decreased range of limb movement [77]. One observation from this study was that while PD had a complex set of symptoms and its measure required a battery of tests [78], where gait was only one factor to be considered, the results showed that the gait variability alone appeared to be suitable for differentiating between case and control. However, this requires extensive investigation before it can be considered for diagnostic purposes.

Another important finding is based on the dependence of gait variability on the walking pattern for individual groups. The results showed that there was a significant increase in the stride interval, stance interval and double support interval variance of gait for straight walking when compared to turns for PD. Literature [79], shows that turning while walking is a challenging task that requires the control of balance. Significant differences in gait variability during turns can be related to Freezing of Gait (FOG) [80] and as an early sign of the progression of disease in PD [81,82]. Age matched control (OL) subjects showed statistically insignificant differences in gait variance, except for double support interval based on walking pattern. Thus, this study confirmed that, gait in PD was disturbed based on the walking pattern. 

This study investigated the GPQI for the groups, which could be used to show how PD gait pattern deviates from normal healthy subjects. The results showed that there was a significant difference in the GPQI value between PD and control subjects. The GPQI value matched with the values reported in [35] for straight walking. Thus, these scores can be used by clinicians to classify the severity of a pathological gait pattern by quantifying the deviation from the control [83] and also to quantify the effect of a treatment or even to evaluate the natural improvement in gait patterns over time [36]. Thus, this study confirmed that, there was a significant difference in gait patterns between the PD and control group.

In conclusion, this study showed that the variance of any of the gait interval parameters obtained using IMU during any of the walking patterns could be used to differentiate between the gait of PD and OL, PD and YL. This can facilitate the quantitative assessment of the patients and can be considered for e-health applications.

## 5. Limitations of This Study

There were two limitations of the present study: Sample size in gender calculation and only the ON-state PD was tested. While 24 PD and 24 age matched controls were a decent size based on literature, this was not sufficient for gender and body size matching, which are factors that contribute to gait parameters. The other factor is that literature shows [84] that there is a significant effect caused by medication, where the difference may be even greater in the OFF state of medication.

## Figures and Tables

**Table 1:** The clinical characteristics in the mean (SD) of three groups–PD, OL, YL.

	PD	OL	YL
Demographic variables			
Age (Years)	71.91 ± 8.64	67.25 ± 3.77	27.91 ± 2.43
Gender (male/female)	17/7	17/7	18/6
Height (cm)	169.26 ± 8.89	166.54 ± 8.20	161.33 ± 4.26
Weight (kg)	81.25 ± 15.86	73.58 ± 12.46	60.29 ± 8.07
Clinical variables			
Disease duration (Years)	4.27 ± 3.15	­	­
UPDRS III	25.69 ± 10.95	­	­
UDysRS	0.79 ± 1.35	-	-
H &Y	2.27 ± 0.94	­	­
Cognitive variables			
Total MOCA score	23.33 ± 5.30	27.33 ± 3.10	28.75 ± 1.35
Visuospatial/executive function	3.5 ± 1.74	4.41 ± 1.13	4.95 ± 0.20
Attention	4.70 ± 1.33	6	6
Delayed recall	2.41 ± 1.97	3.62 ± 1.55	4.16 ± 1.00
Orientation	5.56 ± 0.57	5.95 ± 0.20	5.62 ± 0.71

**Table 2:** The comparison of mean and coefficient of variance of gait intervals for straight with U-turn and turn around a point for each subject group. The *p*-value is tabulated below.

Subject	Walking pattern	µ_st_	µ_sw_	µ_sta_	µ_ds_	σ_st_	σ_sw_	σ_sta_	σ_ds_
PD	Straight walking compared with U-turn	ns	ns	ns	ns	0.001*	ns	0.006*	0.000*
	Straight walking compared with turn around a point	ns	ns	ns	ns	0.002*	ns	0.005*	0.005*
OL	Straight walking compared with U-turn	ns	ns	0.04*	ns	ns	ns	ns	0.021*
	Straight walking compared with turn around a point	ns	0.045*	ns	ns	ns	ns	ns	0.031*
YL	Straight walking compared with U-turn	ns	ns	0.047*	ns	ns	ns	ns	ns
	Straight walking compared with turn around a point	ns	ns	ns	ns	ns	ns	ns	ns
